# Effects of multilevel neuropsychological group intervention (EXAT) in children with executive functioning and attention deficits: a comparative study with typically developing controls

**DOI:** 10.1093/arclin/acag028

**Published:** 2026-04-28

**Authors:** Elina Vierikko, Fiia Takio, Kati Rantanen

**Affiliations:** Psychology Clinic, Faculty of Social Sciences, Tampere University, 33014 Tampere, Finland; Psychology Clinic, Faculty of Social Sciences, Tampere University, 33014 Tampere, Finland; Department of Child Psychiatry, University of Helsinki and Helsinki University Hospital, 00029, Helsinki, Finland; Psychology Clinic, Faculty of Social Sciences, Tampere University, 33014 Tampere, Finland; Psychosocial Support and Medical Rehabilitation, Tampere University Hospital, Wellbeing Services County of Pirkanmaa, 33520 Tampere, Finland; Department of Psychology, Faculty of Medicine, University of Helsinki, 00014 Helsinki, Finland

**Keywords:** executive functions, neuropsychological group intervention, children, intervention effects

## Abstract

**Objective:**

This study examined the effects of the multilevel EXAT (Rehabilitation of Executive Function and Attention) intervention on executive function (EF) in Finnish children with EF and attention deficits, compared to typically developing (TD) controls who received no EF intervention.

**Method:**

The study included 59 children aged 7–12 years with EF deficits and 78 age-matched non-intervention TD controls. The EXAT intervention lasted 9 months and comprised weekly sessions for children, monthly parent sessions, and 2–3 school consultations. Parent-rated EF intervention effects were assessed using the Behavior Rating Inventory of Executive Function (BRIEF). Baseline and post-assessment BRIEF T-scores were compared between groups using t-tests, reliable change indices were calculated for individual EF changes, and linear regression analyses were used to explore associations between background variables and EF changes.

**Results:**

Children in the EXAT group showed significant improvements across all BRIEF indices and subscales, with small to moderate effect sizes. Individual-level analysis indicated that 22%–41% of participants achieved reliable EF improvements, mainly those with severe initial deficits. The control group exhibited minor, clinically insignificant changes.

**Conclusion:**

EXAT positively impacted EF, especially in behavior regulation and metacognition among children with substantial deficits. However, about one-quarter continued to experience EF challenges after the intervention. Findings highlight the importance of individualized assessment and intervention planning within group settings to address diverse EF profiles. Tailoring support to each child’s strengths and weaknesses is essential for optimizing outcomes. Further research with larger, diverse samples and multi-informant assessments is needed to confirm results and examine long-term effects.

## Introduction

Executive functions (EFs) play a fundamental role in children’s cognitive, academic, behavioral, and social development (Zelazo & Carlson, 2020), serving as a top-down control system that regulates thoughts, actions, and emotions (Diamond, 2013; Nigg, 2017). The essential domains of EF—impulse control, Working Memory (WM), and attentional control—are integral to self-regulation (Allan et al., 2015). Consequently, children with EF deficits or delays frequently exhibit higher levels of behavioral problems due to inadequate self-regulation, which affects their emotional, social, and academic competence (Best et al., 2011; Diamantopoulou et al., 2007; Lonigan et al., 2017; Miyake & Friedman, 2012).

EFs develop from childhood through adolescence into early adulthood, exhibiting significant variability among individuals (Best & Miller, 2010; Ferguson et al., 2021; Zelazo et al., 2016). During early childhood (approximately 2–5 years), EF is often described as a unitary construct that begins to differentiate into inhibitory control, WM, and cognitive flexibility across preschool years, becoming more clearly multidimensional through middle childhood and adolescence (Baggetta & Alexander, 2016; Best & Miller, 2010; Blair & Ursache, 2011; Miyake et al., 2000). Although core EF components begin to differentiate during the preschool years, substantial maturation of inhibitory control, WM, and cognitive flexibility continues into the early school years, which is the developmental period relevant to current study (Best & Miller, 2010; Zelazo et al., 2016). These core components underpin complex cognitive functions such as planning, monitoring, and problem-solving (Hofmann et al., 2012; Nigg, 2017). The hierarchical model suggests that difficulties in developmentally earlier and core EF components, such as inhibitory control and WM, can cascade into later-emerging, more complex EF skills (Garon et al., 2008; Zelazo & Carlson, 2020). Consequently, early school years are crucial for interventions aimed at supporting EF development, especially for children showing early signs of EF problems.

EF problems are frequently observed in children with neurodevelopmental and neurological disorders, including Attention-Deficit/Hyperactivity Disorder (ADHD), acquired brain injury, and epilepsy. ADHD, one of the most studied neurodevelopmental disorders, has consistently been associated with elevated rates of EF problems (e.g., Pievsky & McGrath, 2018), although prevalence estimates vary depending on assessment criteria and methodology. In a study examining both group- and individual-level data, Lambek et al. (2011) found that children with ADHD exhibited significantly greater EF problems compared to non-clinical peers, yet only about half met the criteria for EF deficits, highlighting notable heterogeneity within this group. Expanding on this, Kofler et al. (2019) used a criterion-based battery and reported that 89% of children with ADHD showed impairments in at least one EF domain, such as inhibitory control, set shifting, or WM, although only one-third demonstrated deficits across multiple domains. These findings suggest that EF problems may be more prevalent among children with neurodevelopmental disorders than previously estimated. Importantly, children often present with diverse EF profiles and distinct patterns of functional impairment, depending on the nature and severity of their difficulties (Kofler et al., 2019; Roberts et al., 2017). This underscores the need for nuanced assessment practices and individualized intervention strategies that respond to the complexity of EF development in clinical populations.

Earlier studies have investigated various approaches to strengthening EF in children, with cognitive training and holistic intervention strategies emerging as key methods. Cognitive training refers to structured, intensive, and repetitive interventions aimed at alleviating cognitive impairments by targeting core EF domains of WM, inhibitory control, cognitive flexibility, and problem-solving. These tasks are typically delivered through paper-pencil exercises or computerized programs adapted to individual performance levels. Many programs incorporate strategy-learning components that foster metacognitive awareness and facilitate the transfer of acquired skills to everyday contexts, supporting more sustained improvements. The duration and intensity of cognitive training programs for children vary, generally spanning several weeks to a few months, with multiple sessions per week (averaging two to five sessions), each lasting 20–45 mins (see e.g., Laatsch et al., 2020; Karch et al., 2013).

Cognitive training has been applied to both typically developing (TD) children (Eberhart et al., 2025; Scionti et al., 2020) and clinical populations, most commonly children with ADHD (e.g., Chen et al., 2022; Kassai et al., 2019) and acquired brain injuries (Chavez Arana et al., 2023; Laatsch et al., 2007; 2020), with mixed outcomes. Although cognitive training may enhance EF skills in TD children, particularly in areas such as attention, WM, cognitive flexibility, and problem-solving (Kassai et al., 2019), its effects are often modest and may not sufficiently address the broader functional needs of children with neurodevelopmental disorders (Eberhart et al., 2025; Kassai et al., 2019  Scionti et al., 2020). Earlier meta-analyses (e.g., Karch et al., 2013) have highlighted these limitations, especially in children with ADHD. More recent findings suggest that incorporating metacognitive strategies into cognitive training may yield more substantial benefits. For instance, Qiu et al. (2023) found that such training enhances metacognitive skills, contributing to improved organizational functioning in school-aged children with ADHD.

In contrast to focused cognitive training alone, holistic interventions that integrate cognitive, emotional, and social components are suggested to yield more effective and lasting benefits (Diamond & Ling, 2016; Laatch et al., 2020). These approaches build on a multimodal framework that integrates strategies targeting cognition, behavior, emotion, and social functioning, together with structured support for parents and teachers. Evidence from various studies illustrates how integrated approaches can benefit children with different neurodevelopmental and neurological conditions. For example, Miranda et al., (2013) implemented a multilevel EF intervention that included self-instruction techniques for children, behavior modification techniques for parents, and support for behavioral and academic management for teachers, resulting in improvements in visuospatial memory, planning, and reductions in hyperactivity, impulsivity, and inattention. Similarly, Hannesdottir et al. (2017) combined cognitive-behavioral methods with WM training in their group intervention, reporting parent-rated improvements in emotion regulation, social skills, and decreased ADHD symptoms. In the context of acquired brain injury, Laatsch et al. (2020) advocate for individualized treatment plans that incorporate family involvement, direct cognitive training, and technology-enhanced delivery.

Comparing the effects of cognitive training and holistic interventions is challenging because these approaches rely on different types of outcome measures and target different levels of functioning. Cognitive training aims to enhance specific cognitive processes within the child and therefore relies primarily on performance-based EF tasks, which are sensitive to near-transfer effects but show only weak associations with real-world behavior (Melby-Lervåg & Hulme, 2013; Sala & Gobet, 2020; Toplak et al., 2013). Consequently, improvements observed in structured testing situations may not readily translate into functional changes in everyday life. In contrast, holistic interventions aim to influence children’s self-regulation and functioning across home, school, and social environments, and thus typically employ parent and teacher ratings that more accurately capture ecologically meaningful change (Evans et al., 2018;). Their multimodal and context-embedded structure—often involving cognitive, behavioral, emotional, and family components—provides ongoing environmental support and repeated opportunities to practice skills in the settings where they are needed, increasing the likelihood that gains generalize and are maintained over time (Diamond & Ling, 2016; Laatsch et al., 2020). Because the research traditions assess different constructs with distinct methodologies, direct comparisons are challenging and highlight the need for ecologically grounded, multilevel study designs capable of evaluating sustained, real-world improvements in children’s functioning.

This study investigates parent-rated effects of the EXAT (Rehabilitation of EXecutive Function and ATtention) group intervention (Rantanen et al., 2018) on EF in school-aged children with EF and attention deficits. The EXAT intervention, developed at the Psychology Clinic of Tampere University, Finland, is a manualized neuropsychological group program designed to support children aged 6–12 with EF deficits and related behavioral challenges. It is based on a holistic framework that combines cognitive EF training, behavioral strategies, social reinforcement, and psychoeducation to enhance self-regulation and adaptive functioning (Rantanen et al., 2018). By integrating these complementary components, EXAT extends beyond narrow cognitive training and supports the use of EF skills within socially meaningful contexts, thereby enhancing ecological relevance. Furthermore, EXAT is implemented as a multilevel intervention consisting of children’s groups, parents’ groups, and teacher consultations. These coordinated levels work together to strengthen and facilitate generalization of EF skills across everyday environments. Unlike programs focused solely on cognitive training, EXAT emphasizes EF training embedded in peer interaction and active involvement of parents and teachers. This socially grounded and ecologically oriented approach aligns with recommendations for interventions that promote the transfer and application of EF skills to everyday situations.

The present study is part of a larger clinical intervention registry study at the Psychology Clinic of Tampere University, Finland, which was assessed and approved by both the Ethics Committee of University of Tampere and Tampere University Hospital, Pirkanmaa Hospital District. Previous research has demonstrated promising outcomes: a study involving 86 children who participated in EXAT between 2006 and 2013 reported reductions in behavioral symptoms such as impulsivity and restlessness (Rantanen et al., 2018). Another study with a smaller sample of 42 children with epilepsy, ADHD, and EF/attention deficits suggested EXAT may also be effective in addressing EF challenges associated with well-controlled epilepsy (Rantanen et al., 2020). Although these studies primarily focused on behavioral outcomes and ADHD-related symptoms, a detailed examination of the intervention’s impact on everyday EF is lacking.

To address this gap, the current study investigates the effects of the EXAT intervention on EF in school-aged children, comparing outcomes to a TD control group that did not receive any intervention. The study focuses on both cognitive and day-to-day behavioral aspects of EF, with treatment goals tailored to individual EF profiles. By incorporating parent support and school consultation, this research aims to provide empirical evidence on the effectiveness of a multilevel group-based intervention in enhancing EF in real-life contexts (Lee et al., 2022).

The aims were to

1. Study the effects of the EXAT (Rehabilitation of EXecutive Function and ATtention) intervention on parent-rated EF at the group level by comparing children participating in EXAT to non-intervention TD controls. Assessments were conducted at two time points: baseline and post-assessment, over a 9-month study period corresponding to the duration of the EXAT intervention. It is important to note that the control group did not receive the intervention and was assessed at the same time points for comparison.
2. Investigate the clinical significance of individual-level changes in EF within the EXAT and control groups from baseline to post-assessment.
3. Study whether background variables (gender, age, diagnosis, medication, IQ, and learning support at school) are associated with the intervention effects.

Based on the structure and methods used in the EXAT intervention, as well as findings from previous studies (Rantanen et al., 2018; 2020), three primary hypotheses were formulated. First, it was hypothesized that participation in EXAT will result in significant improvements in multiple domains of EF, particularly behavioral regulation (BRI). In the early stages of the intervention, the focus is on strengthening impulse control and BRI, which are essential for establishing a functional and cohesive rehabilitation group. This emphasis is especially critical for children diagnosed with ADHD or exhibiting pronounced ADHD characteristics, as they frequently struggle with inhibitory control and emotional self-regulation. Establishing a functional and cohesive rehabilitation group early on is essential for creating a supportive and effective therapeutic environment. Once these foundational EF skills are established, the emphasis can gradually shift toward training more complex EF. Secondly, following the initial emphasis on behavior regulation, only moderate improvements in metacognitive functions such as WM, planning, monitoring, and organizing are expected. Third, we hypothesized that children in the control group will not exhibit significant changes deviating from normative developmental trajectories between baseline and post-assessment, as they were TD.

## Methods

### The EXAT intervention

EXAT was implemented over a 9-month period (40–42 sessions in total), consisting of weekly 90-min sessions for children in small groups of 4–5 participants, 10 sessions for parents, and 2–3 teacher consultations. The intervention was facilitated by 2–3 licensed neuropsychologists or psychologists with clinical training and formal EXAT certification (25–30 hr), supported by regular supervisory meetings (16–20 hr) with clinically experienced neuropsychologists to ensure treatment fidelity and quality. Group formation, as well as both individual and group-level goals, was informed by a psychological assessment conducted prior to the intervention, alongside parent and teacher reports of everyday EF problems.

In the children’s group, sessions are structured and activities planned according to the rehabilitation goals. Sessions incorporate multimodal methods, such as rehabilitative games, role-play, visual support, and modeling, to promote socially desirable behaviors and practices through behavioral modification and social reinforcement. Activities are adapted as needed, using small subgroups or individualized tasks to address specific skill areas. Clear behavioral expectations are defined to support engagement and skill acquisition. Key components of the cognitive EF training involve teaching general problem-solving strategies and implementing highly structured routines (Mahone & Slomine, 2007). Techniques such as self-instruction programs (Camp & Bash, 1981) and executive scripts (Ylvisaker & Feeney, 2008) are designed to help children articulate their thoughts and develop more effective self-regulation strategies.

In parallel with child sessions, parents attended monthly 90-min support meetings aimed at promoting skill generalization, strengthening parent–child interaction, and providing emotional support. These meetings utilized a variety of methods, including psychoeducation, discussions related to session themes, and homework assignments designed to reinforce the strategies discussed. Parents were provided with psychoeducation about the child’s difficulties in EF and attention, and they were guided to use strategies that support the child’s socially appropriate and expected behavior in everyday situations. The themed discussions allowed parents to share experiences, ask questions, and learn from each other, fostering a supportive community. Homework assignments encouraged parents to practice the strategies at home, facilitating the transfer of skills into everyday situations.

Additionally, 2–3 school consultations were conducted with each child’s teacher to align classroom practices with intervention strategies and reinforce EF development in educational settings. The purpose of the school consultations was to ensure the transfer of methods practiced in rehabilitation, in line with the child’s individual goals, into the child’s everyday life and to strengthen the generalization of these skills, which is regarded as essential for children, as they benefit from consistent support from their environment for skill generalization. The content and themes of the school consultations were not predetermined or scripted. Instead, their content was determined by taking into account the children’s individual progress and needs. Before the consultation, its aims and themes were usually discussed in supervisory meetings.

### Participants

The clinical data consisted of children referred to the EXAT intervention due to confirmed EF deficits at the Psychology Clinic, Tampere University, Finland. Thus, the participants were not recruited for research purposes. The inclusion criteria were age between 7 and 12 years, confirmed EF and attention deficits assessed at referral by local psychologists, and the initiation of the first rehabilitation period, without receiving any concurrent or additional interventions or therapies during the study period. Exclusion criteria included intellectual disability (ICD-10 F70-F79), pervasive developmental disorders (ICD-10 code F84), or other unspecified disorders of psychological development (F88–F89), and psychiatric diagnoses other than ADHD and conduct disorders (F90–F91) (e.g., mood disorders [F30–F39], anxiety disorders [F40–F48]), as well as incomplete or missing parent ratings.

During the years 2013–2017, 93 children aged 6–13 attended EXAT rehabilitation, and their guardians provided informed consent for participation in the study. Of these, 63% met the inclusion criteria and participated in the study, with the sample including 8 participants overlapping with earlier EXAT cohorts (Rantanen et al., 2018; 2020). The final study group consisted of 59 children aged 7–12 years (mean 9.5 years), with a mean Full Scale IQ (FSIQ; Wechsler, 2009; 2010) of 89.5 (ranging from 62 to 128), falling within the low average range of intellectual functioning (Table 1). Analysis of Wechsler index score profiles revealed substantial intra-individual variability across cognitive domains. Approximately 72% of participants demonstrated at least one index score discrepancy of ≥15 points, and nearly half exhibited multiple discrepancies of this magnitude. Discrepancies of ≥23 points, which are considered statistically uncommon in normative samples (Wechsler, 2010), were observed in approximately 28% of cases. The most frequent and pronounced discrepancies involved Working Memory Index (WMI) and Processing Speed Index (PSI), which were significantly lower than Verbal Comprehension Index (VCI) and Perceptual Reasoning Index (PRI) in over half of the sample. In contrast, VCI–PRI discrepancies were less frequent, occurring in approximately one-third of participants.

**Table 1:** Demographic and medical background data of the EXAT study group and the non-intervention typically developing (TD) control group

	EXAT	TD controls	*X* ^*2*^/*t*-test, *p*-value, *d*
*n*	59	78	
Gender (boys/girls)	53/6	38/40	*X^2^* (1) = 25.458, *p* < .001
Age, mean (*SD*) [range]	9.5 (1.4) [7.0–12.6]	9.4 (1.6) [6.9–13.0]	*T* (135) = 0.344, *p* = .732, *d* = 0.059
FSIQ, mean (*SD*) [range]	89.5 (14.7) [62–128]	-	
Verbal Comprehension Index, mean (*SD*) [range]	94.3 (15.3) [64–145]		
Perceptual Reasoning Index, mean (*SD*) [range]	96.5 (16.0) [64–131]		
Working Memory Index, mean (*SD*) [range]	89.7 (13.8) [61–118]		
Processing Speed Index, mean (*SD*) [range]	92.6 (14.1) [67–124]		
WMI, mean (*SD*) [range]	39.7 (5.8)	39.4 (4.9)	*T* (107) = 0.227, *p* = .821, *d* = 0.048
Father’s age, mean (*SD*)	42.4 (7.3)	42.3 (6.4)	*T* (106) = −0.121, *p* = .904, *d* = 0.026
Mother’s education 9–12 years 12–15 years 16 or more Unknown	25 11 23 0	14 22 40 2	*X^2^* (2) = 9.364, *p* = .009
Father’s education 9–12 years 12–15 years 16 or more Unknown	28 10 18 3	30 16 31 1	*X^2^* (2) = 1.627, *p* = .443
Diagnosis (ICD-10)			*X^2^* (2) = 46.526, *p* < .001
No	31 (52.5%)	78	
Yes (ADHD)	21 (35.6%)	0	
Yes (Other^a^)	7 (11.9%)	0	
Medication for ADHD No Yes	38 21 (35.6%)	78 0	*X^2^* (1) = 32.789, *p* < .001
Level of learning support at school			*X^2^* (2) = 53.594, *p* < .001
General support	17 (28.8%)	69	
Intensified support Special support	28 (47.5%) 14 (23.7%)	9 0	
DSM-IV Inattention^b^ mean (*SD*) [range] DSM-IV hyperactivity-impulsivity^b^ mean (*SD*) [range] DSM-IV total^b^ mean (*SD*) [range]	62.9 (10.3) [46–90] 61.6 (10.6) [43–90] 63.6 (9.6) [46–85]	47.6 (5.9) [40–64] 48.6 (6.6) [41–67] 47.9 (5.7) [40–67]	*T* (104) = 9.044, *p* < .001, *d* = 1.894 *T* (104) = 7.298, *p* < .001, *d* = 1.518 *T* (104) = 9.846, *p* < .001, *d* = 2.059

*Note:*  ^a^Other neurodevelopmental disorder *(*ICD–10 codes F80–F89, F91.3), neurological disorder (epilepsy, cerebral palsy) or congenital malformation (fetal alcohol syndrome)

^b^Conners Rating Scales-Revised-L (parents)

*d* = Cohen’s d

All participants in the EXAT group had prominent EF deficits at referral based on clinical neuropsychological assessments conducted by local psychologists. Approximately 36% of participants were diagnosed with ADHD before referral to the EXAT rehabilitation, and 7 children (11.9%) had another diagnosis, including specific or mixed specific developmental disorders, conduct disorder, epilepsy, fetal alcohol syndrome, and cerebral palsy. About 36% were on ADHD medication (Table 1). The session attendance rate varied between 85% and 100% for both the children and the parents. The dropout rate in EXAT is typically low, ranging from 3% to 5% per year.

The control group was selected from a sample of healthy children (*N* = 234, 6–15 years) attending mainstream comprehensive schools. This TD control group was included to provide a culturally and educationally matched reference point for interpreting parent and teacher-rated EF difficulties in the absence of Finnish normative data for the outcome measure used (BRIEF; Gioia et al., 2000). Because BRIEF scores are referenced to U.S. norms, cross-national differences in rating practices may influence score distributions. Including a Finnish control group allowed BRIEF ratings to be contextualized within the same cultural and linguistic environment as the intervention group. The control group was not used to determine intervention eligibility, nor was it intended to serve as a normative Finnish sample; rather, it provided a culturally comparable reference point for interpreting standardized EF ratings.

Non-intervention TD control group consisted of 78 age-matched children without reported severe learning difficulties or EF deficits, with parent assessments returned, and the absence of ongoing rehabilitation for EF deficits was included as controls. Accordingly, children in the control group did not participate in EXAT rehabilitation. None of the controls had a diagnosis of ADHD or were on medication for ADHD symptoms, nor did they receive other therapies. Some children in the control group, however, had mild learning difficulties and received some learning support, for example, remedial instruction. This aligns with the Finnish educational system, where all children are entitled to general learning support, regardless of medical diagnosis (Basic Education Act, 1998). Learning support is organized into three tiers: general, intensified, and special support. General support includes universal strategies and resources within the regular classroom, such as differentiated instruction, remedial teaching, and classroom accommodations. If this proves insufficient, intensified support is provided based on a pedagogical assessment. It is typically temporary and based on tailored learning plans, small group instruction, and closer progress monitoring. Special support is for children with persistent learning difficulties and includes individualized education plans (IEPs), specialized teaching methods, and additional resources, such as special education teachers or assistive technology. Importantly, children may receive intensified or special support based on educational needs alone, without a formal diagnosis.

In this study, nine controls (11.5%) received remedial instruction or part-time special education as part of intensified learning support, compared to 71.2% of the EXAT group participating in intensified or special learning support at baseline (*p* < .001; Table 1). In Finland, remedial instruction refers to targeted instructional support provided within general education to address emerging or persistent academic difficulties. This form of support does not require a formal diagnosis, is not defined as special education, and may be implemented flexibly and for varying durations. For international comparability, this remedial instruction would correspond to the U.S. Response-to-Intervention (RTI) Tier 2 and its most intensive forms to Tier 3 RTI, rather than to IEPs. The TD control group, in turn, is representative of Finnish comprehensive school students, among whom an average of 8.2% received intensified support between 2013 and 2017. Due to the inclusion criteria, there were no controls receiving special support, although an average of 7.4% of Finnish pupils were within this most intensive level of learning support at the time of the study (Official Statistics of Finland, 2018). In addition to the previously mentioned differences in diagnoses and medication, the EXAT and TD control groups also differed in maternal education, with mothers in the control group being more highly educated, and no other differences in background variables were observed (Table 1).

### Measures and procedure

Demographic data and medical history for children attending EXAT were obtained from the Psychology Clinic records. These records were based on parent interviews and referral documentation by local psychologists. Individual psychological test profiles, behavior questionnaires, and clinical reports were reviewed to extract relevant data. Child-level background variables included gender, age, diagnosis, medication, FSIQ, and level of learning support provided at school. Family-level background variables included parental age and education.

Prior to the intervention, all children underwent a comprehensive (neuro)psychological assessment conducted by local psychologists or neuropsychologists. These assessments included interviews and Finnish versions of standardized psychological tests (WPPSI-III, WISC-IV; Wechsler, 2009, 2010, NEPSY-II; Korkman et al., 2008) to evaluate the child’s cognitive profile and EF-related impairments. In addition, parent and teacher behavioral ratings were used. For children with suspected neurodevelopmental or learning difficulties, commonly used standardized behavioral questionnaires in Finland include the Five to Fifteen (5–15R; Kadesjö et al., 2004; Lambek & Trillingsgaard, 2015; see www.5-15.org) and/or the Attention and Executive Function Rating Inventory (ATTEX; Klenberg et al., 2010). The 5–15R is a Nordic norm-referenced, 181- item parent questionnaire assessing eight neurodevelopmental domains in children aged 5–17, including attention, EF, motor skills, perception and language, learning, memory, and emotional/behavioral problems. Interpretation is based on standardized population norms derived from large Danish parent and teacher samples, rather than fixed clinical cut-off scores. Although the 5–15R provides normative percentile thresholds (e.g., the 90th and 98th percentiles) to indicate deviation from age-expected functioning, these thresholds serve as norm-based reference points rather than diagnostic clinical cut-offs. The ATTEX is a standardized teacher report questionnaire assessing everyday attention and EF difficulties in school-aged children. The scale consists of 55 items covering distractibility, impulsivity, activity regulation, and executive control, and it has been standardized on a large Finnish normative sample. ATTEX has demonstrated good reliability, validity, and clinical utility in differentiating EF-related difficulties, including ADHD subtypes (Klenberg et al., 2010).

Only children with confirmed EF and attention difficulties that impair everyday functioning—based on both multidisciplinary clinical assessment and teacher-reported information—were referred to EXAT. Referral decisions did not rely on any single numerical cutoff score. Although the interpreting psychologist used normative indicators such as percentile ranks from the 5–15R and ATTEX in line with standard Finnish clinical practice, these values served only as part of a broader clinical formulation rather than as fixed thresholds for inclusion. Instead, licensed psychologists or neuropsychologists evaluated impairment using a multimethod approach that integrated standardized cognitive test results, parent and teacher-completed behavioral questionnaires, developmental and educational history, clinical interviews, and observations of functioning across home and school contexts. Children were referred when converging evidence across these sources indicated clear, consistent, and contextually meaningful EF and attention-related difficulties that interfered with participation in one or more everyday environments.

This study used a controlled pre-post design, in which the intervention group was assessed before and after EXAT, and a non-intervention TD control group was measured at the same time points. Before the intervention, parents were asked to complete Conners’ Parent Rating Scales — Revised (CPRS-R; Conners, 2001) subscales DSM-IV Inattention, DSM-IV Hyperactivity-Impulsivity, and DSM-IV Total, which were used to determine participants’ attention and a broad range of behavioral problems. Individually converted T-scores (with a mean of 50 and SD of 10) were used to indicate problems within the clinical range. The mean DSM-IV T-scores in the EXAT group were slightly above 60, indicating subclinical ADHD symptoms on average. 35%–48% (*n* = 16–22) of them exceeded the clinical cut-off (T-score ≥65, 1.5 SD), and in 61%–65% (*n* = 28–30) of them, T-scores were above 1 SD. Of the controls, only one child (1.7%) exceeded the clinical cut-off T ≥65, and 2–5 children (3%–8%) exceeded the 1 SD limit.

The primary outcome measure was the parent form of the Behavior Rating Inventory of Executive Function (BRIEF; Gioia et al., 2000), a well-validated instrument for assessing the behavioral aspects of EF in everyday settings. The BRIEF consists of 86 items and was administered twice, at baseline and after a 9-month study period reflecting the duration of the EXAT intervention. It provides an overall score, the Global Executive Composite (GEC), and two broad indices: BRI and metacognition (MI). The BRI includes clinical scales of Inhibition, Shift, and Emotional Control. The MI includes scales for Initiate, WM, Plan/Organize, Organization of Materials, and Monitor. The raw scores of the BRIEF subscales were individually converted into T-scores according to the participant’s gender and age group. The use of age-adjusted T-scores was justified, as they help to identify improvements in EF that go beyond what would normally be expected due to natural maturation. Due to a lack of local normative data, original normative data provided in the manuals were used. The mean T-score is 50, and the standard deviation is 10. In this study, a T-score ≥ 65 (1.5 SD) is considered indicative of clinical-level problems. Additionally, T-scores ranging from 60 to 64 are identified as subclinical problems. Inclusion of children with subthreshold ADHD symptoms is important because these clinically referred children are reported to show a similar level of functional deficits and comorbid symptoms as those meeting full diagnostic criteria for ADHD (Biederman et al., 2018). In this study, internal consistency (Cronbach’s alpha) varied between 0.83 and 0.93 in subscales and 0.95 and 0.97 in indices. Higher T-scores indicate more parent-rated problems of EF in everyday life, thus reflecting lower EF.

At the beginning and end of the rehabilitation, parents were given questionnaires to be completed at home. The primary respondent was the mother, either completing the forms independently or jointly with the father. Completed questionnaires were either returned directly to the Psychology Clinic during a visit or by mail using prepaid envelopes. For children in the control group, parents first received a notification about the study through their child’s teachers. Only those parents who provided written consent participated in the study by completing the questionnaires, which were distributed via the school at corresponding time points, along with written instructions for home completion. These questionnaires were returned by mail using prepaid envelopes.

### Statistical analysis

IBM SPSS Statistics software (version 27.0) was used for statistical analyses. To study the differences between the EXAT and control groups, as well as the effects of the EXAT intervention on parent-rated EF, the baseline and post-assessment T-scores from the BRIEF subscales and indices were used. The difference scores were calculated by subtracting the post-assessment T-score from the baseline-T-score to assess the differences between the EXAT and control groups in the magnitude of EF change during the intervention. *T-*tests were used to assess the statistical significance of differences between the groups.

To assess individual-level changes during the EXAT intervention, that is, to determine whether the change in the BRIEF baseline-to-post-assessment T-scores exceeded what would be expected based on measurement error and reflected a meaningful individual-level change, the reliable change index (RCI; Jacobson & Truax, 1991) was computed for each child. The RCI was calculated by subtracting the post-assessment score from the baseline-score and dividing the result by the standard error of the differences between these two scores. An RCI greater than 1.96 (*p* < .05) indicates reliable change at a 95% confidence level (*p* < .05) rather than measurement error.

Pearson correlation coefficients and Chi-Square tests were used to study the associations between background variables and the difference scores. To assess whether the background variables associated with difference scores predicted EF change, several simple linear regression analyses were conducted to evaluate the extent to which a child’s medication for ADHD could predict the change in EF indices and subscales during the EXAT intervention. *p*-values less than .05 were considered statistically significant. The effect sizes were assessed using Cohen’s d (Cohen, 1988). An effect size of 0.20 indicates a small effect, 0.50 a medium effect, and 0.80 a large effect.

As the final sample consisted of 53 boys and 6 girls in the EXAT group and 38 boys and 40 girls in the TD control group, there was a notable difference in gender distribution between the groups. Due to the overrepresentation of boys in the EXAT group, we conducted an additional analysis to assess the potential impact of this imbalance. Specifically, for each girl in the EXAT group, we selected the closest age-matched girl from the TD group. This subsample of 43 controls included a proportion of age-matched girls (*n* = 5; 13.2%) comparable to that (*n* = 5; 13.2%) as in the EXAT group (11.3%). The statistical analyses were repeated using this matched TD subsample. As the matched sample findings did not differ from the original results, we report the analyses based on the full TD group.

## Results

### The level of parent-rated EF

The EXAT intervention group scored significantly higher (*p* < .001) than the non-intervention TD control group on all parent-rated BRIEF indices and subscales at both baseline and post-assessment (Table 2), indicating more pronounced EF difficulties throughout the study period. Prior to the intervention, 32%–36% of children in the EXAT group exceeded the clinical cut-off scores (BRIEF T-scores ≥65) across all indices (GEC, BRI, MI). A more detailed analysis showed that, depending on the subscale, approximately 20%–39% exhibited clinically significant EF deficits. The highest percentages were observed in WM (about 39%) and Planning/Organizing (about 34%). In contrast, at baseline, only 1.3% of children in the control group exceeded the clinical cut-off scores on the BRIEF indices, and at most 6% exceeded the cut-off on subscales (WM and Organization of Materials). There was more intra-individual variability in the EF profiles in the EXAT group compared to the control group. When applying a threshold of ≥15 T-points, in the control group, large discrepancies between BRI and MI were rare, with only 5.1%. In contrast, the EXAT group demonstrated more heterogeneous EF profiles with 20.3% exhibiting BRI–MI discrepancies.

**Table 2:** Baseline and 9 months post-assessment T-scores for parent ratings of EF (BRIEF – revised) and the proportion (%) of clinically significant EF problems (*T* ≥ 65) in the EXAT intervention group and non-intervention typically developing (TD) control group

		EXAT								Controls					
	**Pre**		**Post**						**Pre**		**Post**				
BRIEF subscales and indices	Mean (*SD*) Range	%	Mean (*SD*) Range	%	*T* ^a^ df	*p*	*d*		Mean (*SD*) Range	%	Mean (*SD*) Range	%	*T* ^a^ df	*p*	*d*
Inhibition	58.56 (10.71) 37–83	25.4	55.78 (10.52) 35–83	22.0	2.447 58	.017	0.319		43.85 (6.56) 36–64	0.0	43.67 (6.29) 36–63	0.0	0.369 77	.713	0.042
Shift	54.93 (13.66) 36–99	23.7	51.37 (10.62) 36–88	8.4	2.313 58	.024	0.301		43.05 (6.95) 36–65	1.3	42.54 (6.31) 36–64	0.0	1.042 77	.300	0.118
Emotional control	56.88 (13.06) 35–80	30.5	53.47 (12.32) 36–88	20.3	2.878 58	.006	0.375		45.91 (8.34) 36–73	3.9	45.26 (7.58) 36–68	1.3	0.906 77	.368	0.103
Initiate	56.12 (11.92) 38–82	20.3	54.81 (10.77) 35–81	20.3	1.085 58	.282	0.141		46.45 (7.32) 35–68	1.3	46.79 (6.87) 35–63	0.0	−0.922 77	.359	−0.104
Working memory	60.69 (11.29) 35–78	39.0	59.73 (10.24) 38–80	32.2	0.967 58	.337	0.126		47.19 (9.29) 35–72	6.4	47.46 (10.15) 19–74	6.4	−0.445 77	.658	−0.050
Planning/organize	56.64 (10.81) 37–80	33.9	54.27 (10.37) 33–76	18.6	2.048 58	.045	0.267		44.33 (8.64) 33–86	2.6	45.28 (9.07) 33–86	2.7	−2.065 77	.042	−0.234
Organization of materials	58.08 (9.79) 32–71	30.5	56.00 (10.24) 33–71	24.1	2.187 58	.033	0.285		47.41 (9.63) 32–69	6.4	48.18 (9.56) 32–72	7.7	−1.305 77	.196	−0.148
Monitoring	59.29 (9.94) 39–78	30.5	56.36 (11.67) 34–78	25.9	2.339 58	.023	0.305		42.55 (7.98) 28–69	2.6	42.96 (8.55) 28–70	2.6	−1.108 77	.271	−0.125
BRI	58.00 (11.73) 36–86	32.2	54.42 (11.33) 35–86	22.0	2.785 58	.007	0.363		44.68 (7.41) 35–70	1.3	43.23 (6.79) 35–63	0.0	0.723 77	.235	0.082
MI	60.08 (11.12) 38–96	35.6	56.49 (10.19) 31–78	22.8	3.211 58	.002	0.418		43.83 (7.21) 31–74	1.3	45.41 (8.07) 31–64	0.0	−1.668 77	.050	−0.189
GEC	60.14 (10.26) 40–81	33.9	56.75 (10.81) 32–84	26.3	3.200 58	.002	0.417		43.85 (6.56) 32–74	1.3	44.04 (7.02) 31–63	0.0	−0.434 77	.333	−0.049

Note: BRI= Behavioral Regulation Index, MI = Metacognition Index, GEC = Global Executive Composite.

^a^  *T*- test, *d* = effect size Cohen’s *d*, % = the proportion of clinically significant EF problems (*T* ≥ 65).

### Group-level EF changes in the EXAT and non-intervention TD groups

After the intervention, parents in the EXAT group reported a statistically significant decrease in all BRIEF indices and subscale scores (Table 2), as well as a reduction in the proportion of clinically significant EF deficits, except for Initiate and WM. Based on the BRIEF indices (GEC, BRI, MI), the proportion of clinically significant EF problems decreased by approximately 8% (from 32%–36% to 22%–26%). Although most indices and subscale scores showed significant baseline–post-assessment changes in the EXAT group, effect sizes were small or non-significant (Table 2).

**Figure 1:**
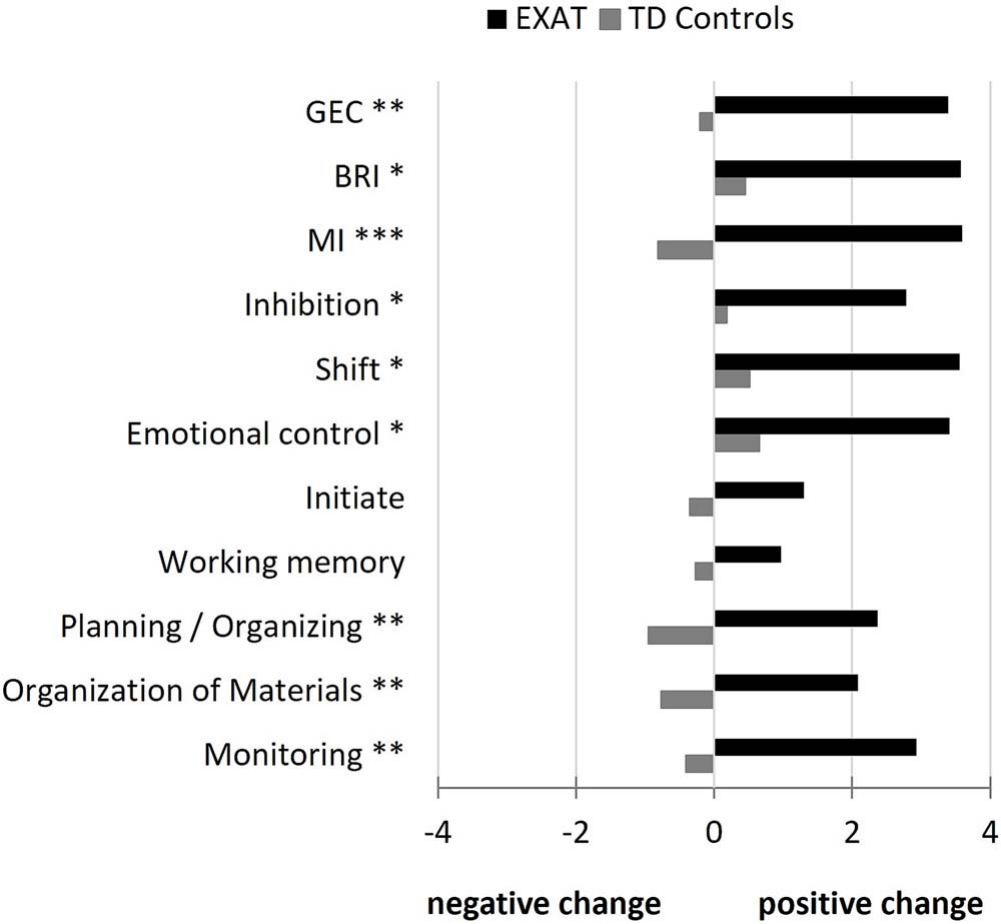
Baseline-post assessment-difference scores in the BRIEF indices of Global Executive Composite (= GEC), Behavioral Regulation (= BRI), and Metacognition (= MI), and subscales in EXAT intervention group and non-intervention typically developing (TD) controls. A positive difference score indicates decreased difficulties at post-assessment compared to baseline. ^*^p < 05, ^**^p < .01.

In the non-intervention TD control group, no significant changes were observed between baseline and post-assessment scores over the 9-month study period, except for MI subscale Plan/Organize, which showed a small increase in scores (Table 2). At most, about 8% of control children exhibited clinically significant problems during this period, resulting in negligible effect sizes. Baseline–post-assessment difference scores (Fig. 1) were positive across all EF domains in the EXAT group, indicating decreased EF problems, and were significantly larger than in the control group for all subscales except for Initiate and WM, where no statistically significant group differences were observed.

### Individual-level EF changes in the EXAT and non-intervention TD groups

Individual-level changes were examined using the RCI. The proportion of children showing positive or negative RC varied across BRIEF indices, subscales, and groups (EXAT and non-intervention TD controls; Table 3). In the EXAT group, a higher proportion showed positive RC across indices and subscales compared to controls. Most children in the TD group showed no clinically reliable change, whereas in the EXAT group, a substantial proportion exhibited either positive or negative RC, depending on the domain. Negative RC was more common in the EXAT group than in controls, but still less frequent than positive change. Detailed percentages are presented in Table 3.

**Table 3:** The number and proportion (%) of children in the EXAT and non-intervention typically developing (TD) control group demonstrating reliable positive (RC+) or negative (RC−) change and no change (RC0) in EF based on the Global Executive Composite (GEC) from baseline to post-assessment during the study period, reported at the 95% confidence level (RCI > 1.96)

	EXAT			TD controls		
BRIEF indices and scales	RC+ *n* (%)	RC0 *n* (%)	RC- *n* (%)	RC+ *n* (%)	RC0 *n* (%)	RC- *n* (%)
GEC	14 (23.7)	44 (74.6)	1 (1.7)	2 (2.6)	74 (94.9)	2 (2.6)
BRI	17 (28.8)	35 (59.3)	7 (11.9)	5 (6.4)	71 (91.0)	2 (2.6)
MI	16 (27.1)	40 (67.8)	3 (5.1)	1 (1.3)	71 (91.0)	6 (7.7)
Inhibition	20 (33.9)	30 (50.8)	9 (15.3)	4 (5.1)	71 (91.0)	3 (3.8)
Shift	24 (40.7)	20 (33.9)	15 (25.4)	5 (6.4)	69 (88.5)	4 (5.1)
Emotional control	24 (40.7)	27 (45.8)	8 (13.6)	4 (5.1)	70 (89.7)	4 (5.1)
Initiate	21 (35.6)	24 (40.7)	14 (23.7)	3 (3.8)	68 (87.2)	7 (9.0)
Working memory	13 (22.0)	35 (59.3)	11 (18.6)	5 (6.4)	69 (88.5)	4 (5.1)
Planning/organizing	21 (35.6)	28 (47.5)	10 (16.9)	2 (2.6)	68 (87.2)	8 (10.3)
Organization of materials	20 (33.9)	30 (50.8)	9 (15.3)	4 (5.1)	65 (83.3)	9 (11.5)
Monitoring	22 (37.3)	24 (40.7)	13 (22.0)	3 (3.8)	70 (89.7)	5 (6.4)

Note: GEC = Global Executive Composite, BRI= Behavioral Regulation Index, MI = Metacognition Index.

**Figure 2:**
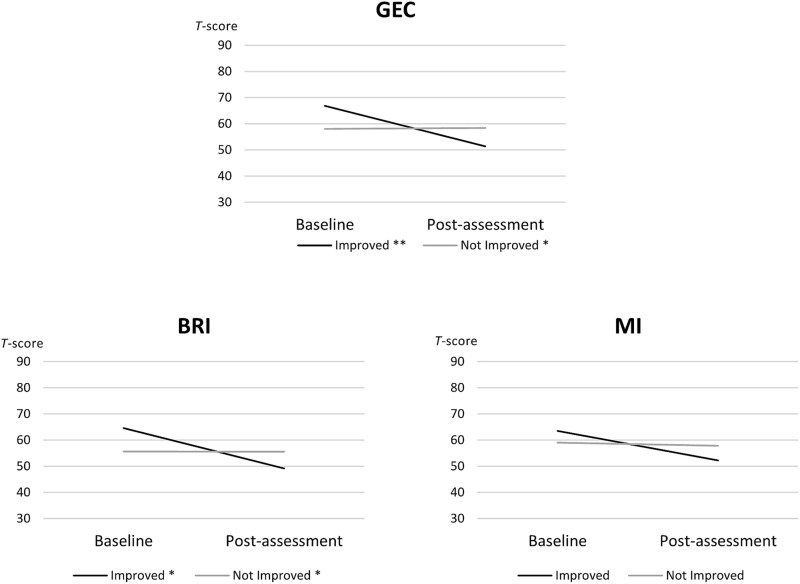
BRIEF T-score differences between children who showed a positive reliable change (RC) in the Global Executive Composite (GEC; *improved*) and those who showed a negative or nonsignificant RC (*not improved*), including both the EXAT and non-intervention typically developing (TD) control groups, across the GEC, Metacognition Index (MI), and Behavioral Regulation Index (BRI). ^*^p < .05, ^**^p < .01.

Figure 2 illustrates baseline–post-assessment BRIEF index difference scores within the EXAT group only, comparing children who improved based on a positive RC in GEC (*n* = 14, defined as Improved group) and those who did not (non-significant RC, *n* = 44; negative RC, *n* = 1, defined as Not Improved group). The Improved group initially had more problems on the BRI at baseline than the Not Improved group. At post-assessment, parent-rated problems in the Improved group significantly decreased, falling below the baseline levels of the Not Improved group. A similar trend was observed in MI, but the reduction was smaller and not statistically significant.

Within the EXAT group, children in the Improved group had higher BRIEF T-scores across all indices and subscales, indicating greater EF problems at the beginning of the intervention compared to the Not Improved group. At the beginning of the intervention, statistically significant differences were observed between the Improved and Not Improved groups in the GEC and BRI indices, particularly in the subscales of Inhibition, Shift, and Initiate. The Improved group showed more pronounced difficulties in these areas. After the intervention, children with a positive RC in the GEC showed fewer EF problems compared to those with nonsignificant or negative RC. The mean post-assessment T-score difference between the Improved and Not Improved groups was approximately 7 points. However, statistically significant differences between the groups were found in the BRI, WM, and Monitoring, where children in the Not Improved group had higher T-scores, indicating more pronounced difficulties in these specific areas. No differences were found between the groups in background variables.

### Background variables associated with the EF change

Within the EXAT intervention group, none of the continuous background variables or ADHD symptoms measured with the CPRS DSM-indices before EXAT correlated statistically significantly to EF difference scores of the BRIEF. Of the categorical background variables, only the child’s ADHD medication was related to EF difference scores in MI in EXAT group. In children with ADHD medication, the mean baseline–post-assessment difference score was greater than in children without medication (*T* = −2.21, *df* = 55, *p* = .031). Simple linear regression analysis was conducted to evaluate the extent to which child’s medication for ADHD predicts the change in MI during the EXAT intervention. The overall regression model was statistically significant (*F* (1.57) = 4.267, *p* = .043). The *R^2^* (0.070) indicated that ADHD medication explained approximately 7% of the variance in MI (*β* = 4.698, *p* = .043).

## Discussion

The present study examined the effects of the multilevel EXAT intervention on parent-reported EF in clinically referred school-aged children with confirmed EF and attention deficits. Intervention effects were analyzed at both the group-level, relative to a non-intervention TD control group, and at the individual-level using clinically significant change indices.

At the group level, the EXAT intervention resulted in statistically significant improvements in BRI, including inhibition, flexibility, and emotional control, as well as in some metacognitive processes such as planning, organization, and monitoring. Although these improvements were statistically significant, effect sizes remained small, indicating a modest overall impact on everyday EF. In contrast, initiation and WM did not show significant changes. As expected, the non-intervention TD control group remained within the normative range across the study period, with only minor, clinically insignificant changes in planning and organizational skills.

At the individual level, approximately 22%–41% of the children demonstrated clinically significant improvement, most often those who exhibited pronounced baseline difficulties in BRI. These children showed broad gains across EF domains. In contrast, children who did not improve remained relatively unchanged and, by the end of the intervention, displayed greater difficulties in BRI, WM, and monitoring relative to the improvers. This variability highlights the importance of considering individual profiles when interpreting intervention outcomes in heterogeneous clinical samples.

The present group-level findings align with previous research indicating that multilevel and holistic interventions can improve EF, particularly in BRI and MI (Diamond & Ling, 2016; Laatsch et al., 2020; Miranda et al., 2013). Integrating cognitive, behavioral, and social strategies, as implemented in EXAT, appears to foster meaningful changes in everyday functioning (Evans et al., 2018; Takacs & Kassai, 2019). This study also extends earlier research on EXAT (Rantanen et al., 2018; 2020), which primarily focused on behavioral outcomes such as reduced impulsivity and restlessness, by providing a detailed analysis of parent-rated everyday EF functioning. In line with previous findings, the current results demonstrate improvements in BRI, most notably in emotional control, flexibility, and inhibition. These findings further support effects of EXAT in addressing core self-regulatory challenges in children with EF and attention deficits.

Importantly, the present study also highlights gains in metacognitive domains, including planning and monitoring. Whereas earlier studies reported general behavioral improvements, the current results suggest that EXAT may also support higher-order EF processes.

This broadens the understanding of the intervention’s impact and suggests potential for wider cognitive and everyday functional benefits. These findings are particularly relevant during the early school years, a critical developmental period for core EF components (Garon et al., 2008; Zelazo & Carlson, 2020), making this phase especially conducive to interventions aimed at enhancing EF skills. The EXAT intervention, which initially targets impulse control and self-regulation, aligns well with the needs of children during this transition, as these skills are essential for academic success, including maintaining focus and following instructions (Poutanen et al., 2016). Recognizing and addressing difficulties in these core EF areas early is crucial, and the present findings emphasize the importance of timely, targeted support during this sensitive developmental stage.

The small change in the TD group’s Plan/Organize scores remained modest and within the normative range, consistent with typical test–retest variability in parent-rated EF (Gioia et al., 2000). Both groups experienced rising academic expectations, which could plausibly contribute to minor fluctuations in everyday planning; however, only the EXAT group showed broad and consistent improvements across all EF domains. This pattern suggests that EXAT-related gains exceed what would be expected from normal developmental variation or contextual pressures, whereas the small TD group changes likely reflect typical variability over a nine-month period. In addition, in EXAT, teacher consultations and parent-focused guidance may have increased the consistency of adult support across the child’s daily environments, which in turn may have contributed to the reductions in EF difficulties observed in the EXAT group.

Marked heterogeneity in baseline EF profiles in the EXAT group, typical for clinically referred samples, likely contributed to the mixed outcomes at both group and individual levels. Children differed in neurodevelopmental profiles, severity of EF deficits, and contextual factors such as family engagement and comorbidity. Those with milder initial difficulties had limited room for measurable improvement, and the conservative, reliable change threshold may have underestimated smaller but clinically relevant gains. Profile-level analyses further indicated greater intra-individual divergence between BRI and MI among the EXAT participants than TD children, suggesting uneven EF patterns that may correspond to distinct underlying mechanisms. Cognitive profiles followed a similar pattern, with lower FSIQ driven primarily by weaker WM and processing speed—consistent with prior findings in children with ADHD and EF deficits (Calub et al., 2019; Pievsky & McGrath, 2018). Such domain-specific variability may moderate responsiveness to intervention and underscores the need for individualized interpretation of outcomes.

Individual variability in the effectiveness of cognitive and holistic interventions for children with EF deficits has been widely documented, reflecting differences in baseline profiles, intervention components, and contextual factors (Diamond & Ling, 2016; Eberhart et al., 2025; Kassai et al., 2019; Laatsch et al., 2020; Scionti et al., 2020). Within this context, comparing children who showed reliable improvement with those who did not provide insight into intervention responsiveness. Children who improved as expected had greater EF difficulties at baseline, particularly in BRI domains such as inhibition, shifting, and planning. These findings are consistent with our initial hypothesis that EXAT would be effective in enhancing behavioral self-regulation. This emphasis is reflected in the intervention structure, where early sessions focus on strengthening BRI to establish effective group functioning and support learning. This approach aligns with theoretical perspectives highlighting the central role of inhibitory control and related processes in EF (Diamond, 2013). Managing impulsivity and inhibition early appears critical for participation in group intervention, which may partly explain why improvements were most evident in these domains.

The EXAT intervention did not yield significant improvements in WM or initiation, suggesting that these domains may require more targeted or intensive strategies. Previous research indicates mixed findings regarding WM training, with improvements often limited to specific tasks without generalization (Melby-Lervåg & Hulme, 2013; Sala & Gobet, 2020). Although EXAT included cognitive components and lasted longer than typical cognitive training programs, early sessions prioritized BRI. Initiation was practiced in both individual tasks and group activities, reflecting a metacognitive focus; however, the level of practice may not have been sufficient to produce measurable gains. WM was not directly targeted, particularly during the initial phase, which may further explain the absence of significant improvements. These findings suggest that integrating targeted WM practice into broader, contextually rich intervention frameworks may enhance outcomes, particularly when combined with sufficient intensity and individualization.

The EXAT model incorporates individualization within a group-based framework. Psychological assessment prior to participation informs planning and ensures that interventions address diverse needs. This flexibility may be particularly relevant for children with atypical EF trajectories, such as those with ADHD. Further research should examine age-related effects and optimal timing for EF interventions, building on Diamond’s (2013) emphasis on early, adaptive strategies tailored to unique developmental profiles.

### Limitations

This study employed a simple pre-post-design without a wait-list control group, which limits causal inferences. The small sample size further restricts the generalizability of the findings.

Participants were clinically referred for neuropsychological assessment because of suspected attention, EF, or school-related difficulties. As such, the sample may be considered broadly representative of children who are typically referred for rehabilitation due to functional challenges in daily life. Further, this study was conducted in Finland, a country characterized by universal access to healthcare and education. This context may affect the feasibility of implementing the EXAT intervention and limit the generalizability of the findings to countries with different systemic frameworks, resource availability, or intervention practices.

A major limitation is the reliance on parent-reported questionnaires as the only outcome measure. This single-informant approach may not capture the multifaceted nature of EF difficulties across different contexts and is susceptible to parental bias, potentially overestimation of treatment effects (Sonuga-Barke et al., 2013). Furthermore, teacher-rated EF data were unavailable due to low response rates, often related to limited familiarity with the child and time constraints, which restricts the generalizability of the findings to the classroom context. To improve the validity of EF assessments, future research should incorporate multi-informant approaches and objective measures (Toplak et al., 2013). Although neuropsychological tests offer insights into cognitive performance in structured settings, they may not reflect everyday functioning. Conversely, behavioral ratings provide perspectives on real-world EF deficits, allowing for a better understanding of how these difficulties manifest in daily life, but are vulnerable to bias. A combined approach can yield a more comprehensive understanding of treatment effects, including subtle or subclinical changes, and support more individualized intervention strategies.

Interpretation of the findings is also constrained by limitations in test availability and the absence of country-specific norms. In Finland, as in other small countries with limited language areas, slow adaptation and standardization of psychological tests lead to reliance on older measures, reducing measurement precision. In this study, EXAT referrals were based on comprehensive clinical judgment and functional impairment across contexts rather than fixed test score cutoffs, and thus, the absence of updated norms did not influence participant selection. For example, the original BRIEF (Gioia et al., 2000) lacks Finnish norms and may yield score patterns that differ from U.S. expectations. To partly address this, a TD control group was included to provide a culturally relevant reference point; although not a normative sample, their lower T scores likely reflect cultural or educational differences in rating practices rather than true EF differences. These issues underscore the need for updated, culturally appropriate EF measures for Finnish school-aged children, and future work would benefit from the use of more recent tools such as the BRIEF2 (Gioia et al., 2015).

Finally, the unequal gender distribution between the EXAT and TD groups represents an additional limitation, as the EXAT group was predominantly male. ADHD and attentional difficulties are more frequently diagnosed in boys, which may reflect genuine differences in symptom severity. However, gender-based behavioral expectations can also influence parental reporting. Rucklidge (2010) notes that parents and teachers tend to notice and report boys’ hyperactive and impulsive behaviors more readily, although inattentive symptoms in girls are often overlooked or misinterpreted as shyness or daydreaming. Although supplementary analyses using a gender-adjusted TD subsample yielded consistent results, the possibility of gender-related bias cannot be entirely ruled out. Future studies should consider stratified sampling or include gender as a covariate to better account for these effects.

Despite these limitations, the study has several notable strengths. First, the EXAT intervention addresses a clinically important topic within neuropsychological practice, focusing on children with significant EF and attention deficits. The study sample reflects the heterogeneity typical in real-world clinical populations, including comorbid conditions and diverse EF profiles, which enhances the ecological relevance of the findings. Another strength is the individualized examination of RCI, which allows for a nuanced understanding of intervention effects beyond group-level analyses. This approach highlights the variability in outcomes among individual participants, emphasizing the need for tailored strategies in clinical practice.

The present findings also offer tentative implications for the implementation of multilevel EF interventions in practice. In Finland, EXAT has been adopted as a form of medical rehabilitation in several hospital districts and Wellbeing services counties, and clinicians nationwide have received training in its delivery. Because EXAT combines child group sessions with parent groups and teacher consultations, it can be implemented relatively cost-efficiently and adapted flexibly to settings—such as clinics and schools—where collaborative support structures exist. The model may be particularly relevant for primary school-aged children whose EF-related difficulties affect across multiple environments and for families who benefit from structured psychoeducation and peer support. Broader implementation, however, would require local coordination between health care and education systems, and local adaptation may influence feasibility. Nonetheless, the multilevel and ecologically oriented structure of EXAT aligns with current recommendations for supporting children with complex EF needs.

## Conclusion

EXAT shows promise as a structured yet holistic intervention that combines cognitive EF training, behavior strategies, and collaboration with parents and teachers. The results underscore the importance of individualized, profile-based planning within group programs to address diverse needs. Future research using randomized or wait-list–controlled designs, larger and more diverse samples, multi-informant and performance-based outcomes, and longer follow-up is needed to clarify moderators, durability, and mechanisms of change.
